# A Change-Management Approach to Closing Care Gaps in a Federally Qualified Health Center: A Rural Kentucky Case Study

**DOI:** 10.5888/pcd16.180589

**Published:** 2019-08-08

**Authors:** Angela L. Carman, Robin C. Vanderpool, Lindsay R. Stradtman, Emily A. Edmiston

**Affiliations:** 1 University of Kentucky College of Public Health, Department of Health, Behavior & Society, Lexington, Kentucky

## Abstract

Effective organizational change requires intentional planning. We applied Kotter’s 8-Step Process for Leading Change model in understanding and evaluating how a federally qualified health center in rural Kentucky implemented a significant organizational change — a proactive office encounter (POE) model — to improve preventive care service delivery, close care gaps, and reduce health disparities among its patients. We completed qualitative interviews with 21 clinic personnel (eg, administrators, physicians, support staff, care coordinators) who were directly involved with POE implementation. We found evidence of steps 1 through 7 of Kotter’s 8 steps of change in the POE implementation process. Step 8, anchoring new approaches in the organizational culture, was an area for improvement. Change-management models, such as Kotter’s 8-Step Process for Leading Change, provide a systematic guide for health clinics to implement sustainable organizational change aimed at improving patient health outcomes.

**Summary**

**What is already known on this topic?**

Delivery of quality adult preventive services, such as immunizations, cancer screening, tobacco use assessment, and cholesterol screening, is essential to improving population health. However, gaps often exist between recommended best practices in delivering preventive services and the care that is actually delivered.

**What is added by this report?**

Use of change-management approaches can help guide organizations through strategic implementation and sustainability of an evidence-based intervention, such as a Proactive Office Encounter model, to close the gap between recommendations and delivery.

**What are the implications for public health practice?**

The change-management approach not only serves to improve clinical workflows of an organization but also serves to improve patient outcomes and, subsequently, population health.

## Introduction

Delivery of quality adult preventive services, such as immunizations, cancer screening, tobacco use assessment, and cholesterol screening, is essential to improving population health (1). However, gaps often exist between recommended best practices in delivering preventive services and the care that is actually delivered (2). To effectively close care gaps in health care delivery environments, organizational changes may be needed to improve clinic culture, staff workflow efficiency, information technology supports (eg, electronic medical records), and mechanisms for continuous quality improvement. In particular, it is vital to assess *how* these changes are introduced and maintained within an organization. Research on organizational culture suggests organizations are “living, social organisms” and that change, without intentional planning and reinforcement, will not last (3). In addition, if change is not managed intentionally “over the intermediate and long terms, the old ways begin to creep back in” (3). Additional research suggests that change has both situational and psychological aspects, and ignoring either will result in a nonsustainable situation, wherein the organization is in a constant cycle of change implementation without achieving desired results (4,5).

Kotter’s 8-Step Process for Leading Change, a well-known change-management model, posits that situational and psychological aspects of change are addressed through a series of dynamic, nonlinear steps (4,6) (Box). The 3 main tenets of Kotter’s model are creating a climate for change, engaging and enabling the whole organization, and implementing and sustaining change (7). The theory helps to identify errors made by organizations as change is introduced and counteract people who are ill-prepared and processes that are not designed to manage and sustain it (6). Kotter’s model has been widely studied in business (8) and has also been applied in the health care environment. One health care application of Kotter’s model was an assessment of organizational changes for improving patient safety, in which the model was deemed highly effective as a framework for guiding change (9). Other examples of using Kotter’s model to implement new initiatives are an implementation of healthy workplace initiatives, an integration of electronic medical records into clinical practice, and an improvement of clinical and provider communication practices (8–11).

**Box. The 3 Tenets and 8 Steps of Kotter’s 8-Step Process for Leading Change (4,6)**

**Tenet 1: Creating a climate for change**

Step 1: Establishing a sense of urgency

Step 2: Creating a guiding coalition

Step 3: Developing a vision and strategy

**Tenet 2: Engaging and enabling the whole organization**

Step 4: Communicating the change vision

Step 5: Empowering employees for broad-based action

Step 6: Generating short-term wins

**Tenet 3: Implementing and sustaining change**

Step 7: Consolidating gains and producing more change

Step 8: Anchoring new approaches in the culture

## Purpose and Objectives

This case study focused on a retrospective application of the change-management model to understand how a federally qualified health center (FQHC) in rural Kentucky implemented a significant organizational change to improve its delivery of preventive care services, close care gaps, and reduce health disparities among its patient population. The White House Clinics, an 8-site FQHC in Appalachian Kentucky, implemented an evidence-based model, the proactive office encounter (POE) (12). POE was originally developed by Kaiser Permanente Southern California Region to systematically identify preventive care gaps through the strategic use of organizational workflow changes, refinements in information technology, and continuous quality improvement (13). The POE model requires that patients’ medical records are assessed before all visits, so that the clinic is prepared for the original purpose of the visit and to ensure that patients adhere to preventive care (12). This case study evaluated the implementation of POE to determine whether the process aligned with Kotter’s model, theorizing that use of these steps could increase the likelihood of POE sustainability in the FQHC.

## Intervention Approach

Faculty and staff members of the University of Kentucky College of Public Health collaborated with White House Clinics to evaluate their implementation of POE and alignment with Kotter’s model. The overall goal was to determine whether POE implementation at White House Clinics could be considered a successful organizational change. One of the primary evaluation activities was a series of qualitative interviews with clinic personnel directly involved with POE implementation. These personnel, or “key change agents,” included clinic administrators (ie, chief executive officer, chief operating officer); physicians (eg, medical director, clinic leaders); paraprofessionals, nurses, and certified medical assistants providing direct patient care; and care coordinators charged with reviewing patients’ medical records for preventive care gaps. We approached 22 key change agents for interviews and 22 agreed to participate; however, 1 person was unavailable to complete the interview because of a scheduling conflict. Therefore, 21 key change agents completed interviews. The semistructured interview guide was informed by organizational theory (14), Kotter’s model (7), and the interdisciplinary expertise of the investigative staff (eg, implementation science, public health practice, health care administration).

### Interview content

Kotter describes the first tenet, creating a culture of change within an organization, as 1) establishing a sense of urgency, 2) creating a guiding coalition, and 3) developing a vision and strategy (7). We asked participants to describe the extent to which implementing POE was an important priority at the White House Clinics, compared with other priorities, and how frequently POE implementation was discussed with staff members to determine a sense of urgency for the project. To determine whether the organization had used a guiding coalition structure to lead the project, we asked participants about whom they perceived to be POE implementation leaders.

The second tenet, engaging and enabling the whole organization, is described as 1) communicating the change vision, 2) empowering employees for broad-based action, and 3) generating short-term wins (7). We asked participants about resources used to implement POE, unanticipated challenges or barriers, and how the implementation could have been made easier.

Kotter describes the methods of the third tenet, implementing and sustaining change, as 1) consolidating gains and producing more change and 2) anchoring new approaches in the culture (7). We asked participants how implementation of POE affected workload and what steps were taken to make POE part of the daily routine.

We conducted interviews in July 2015 through September 2016. They lasted approximately 1 hour and were audio-recorded with the participant’s permission. Participants were given a $75 gift card as a token of appreciation.

## Evaluation Methods

We transcribed and analyzed interview recordings by using theoretical coding with constant comparative techniques (15) to align participants’ thoughts and comments with Kotter’s model. First, we ensured that team members understood the operational definitions of Kotter’s model. We then categorized participant comments according to relevant steps of the Kotter framework, and we discussed findings until consensus was reached. We organized our findings according to Kotter’s 3 major tenets and 8 steps. The University of Kentucky Institutional Review Board approved all study procedures.

## Results

Most participants were non-Hispanic white (n = 19), female (n = 18), and aged 50 or younger (n = 18) (Table). They represented a range of positions within the organization and had been with White House Clinics for an average of 7 years.

**Table:** Characteristics of Interview Participants (N = 21) in a Qualitative Study of the Implementation of the Proactive Office Encounter^a^ at an 8-Site Federally Qualified Health Center in Kentucky and Its Alignment With Kotter’s 8-Step Process for Leading Change Model^b^

Variable	No. of Participants
**Sex**	
Male	3
Female	18
**Age, y**	
20–30	4
31–40	5
41–50	9
51–60	1
61–70	2
**Race**	
Non-Hispanic white	19
Asian	1
American Indian or Alaska Native	1
**Length of time working at federally qualified health center, y**	
≤2	2
3–5	7
6–10	5
≥11	4
**Job responsibility**	
Chief executive officer	1
Enabling services manager	1
Provider (family practice, pediatrics)	6
Nursing staff (registered nurse, registered medical assistant, certified medical assistant)	5
Team leader	3
Patient care coordinator	5

a An evidence-based model originally developed by Kaiser Permanente Southern California Region to systematically identify preventive care gaps through the strategic use of organizational workflow changes, refinements in information technology, and continuous quality improvement (13).

b A change-management model that posits that situational and psychological aspects of change are addressed through a series of dynamic, nonlinear steps (4,6).

### Creating a climate for change

Overwhelmingly, participants described POE implementation as one of the organization’s highest priorities. For example, a physician stated, “The push from administration to keep this project going was important. This was not something we could let fail.”

In response to the question about a guiding coalition and POE implementation leadership, most participants stated that the chief executive officer was the primary leader; the enabling services service line manager and the medical director were also named. These leaders were confirmed by the chief executive officer to be the team members initially involved in POE implementation.

### Engaging and enabling the whole organization

One physician described organizational communication by stating, “There were probably tons of meetings just to understand what it [POE] means, what it entails, what we have to do and how to do it.” Some meetings were focused on building employee skills, such as new standing orders, workflow changes, motivational interviewing techniques, and protocols for “huddle meetings,” where clinicians and support staff members review the day’s medical records and patient list.

Interviews revealed examples of empowering employees for broad-based action. Standing orders, which identify clinical care processes that can be completed by clinical support staff members under stated conditions, were updated. One physician said, “This project told the nurses ‘you can do this without asking the provider.’ Certain immunizations, for example. This project empowers the nurse to do things. That’s been a big change.”

Short-term wins were seen as the organization reached milestones necessary for full implementation of POE. Examples of milestones included hiring new staff members (eg, care coordinators), development of health maintenance forms, and training providers and staff members who act on the information contained in the forms. In addition, White House Clinics leadership invited their academic partners to provide training on organizational communication strategies for the new processes. One of the most exciting wins, as described by the chief executive officer, was the increase in the number of patients receiving recommended preventive screenings such as mammograms, colonoscopies, and hepatitis C and HIV testing. In addition, the chief executive officer used the timing of these milestones as an opportunity to show gratitude to the staff who were instrumental in implementing POE: “We are glad you are here, your work is important. I know this is overwhelming right now, but we are really glad that you are doing it.”

### Implementing and sustaining change

The impact of POE on the clinic staff was evident in statements from care coordinators and nurses. One care coordinator said, “At first it [POE] greatly increased my workload, but now my workload has been reduced.” A nurse said, “It helps provide better care because you’re getting stuff done that you would otherwise forget to do.” A pediatrician provided an example of how the organization built on the initial POE implementation with adult patients to produce additional change: “We are changing the [health maintenance forms] process for pediatric patients and it’s going to be completely different.” The changes included a greater focus on the scheduling frequency of well-child examinations and mapping screening tests such as hearing, vision, and dental examinations; human papillomavirus vaccination; and tobacco screening to children in the appropriate age group.

Interviews revealed instances where leadership worked to anchor the preventive focus that POE brings into the clinic culture. Through training and meetings with leadership, staff members indicated a common understanding that POE would be the vehicle to ensure patients proactively receive preventive care and increase the likelihood of early detection of health issues. Standard use of health maintenance forms and morning huddle meetings were instituted. In addition, the chief executive officer said that after POE implementation, salary structure adjustments were made so that all staff positions were paid from the same wage scale. These adjustments broke down the informal hierarchy that previously placed care coordinators in a lower position than clinical support staff members. She stated emphatically, “You aren’t going to tell me that what the scrubbers [care coordinators] do is not contributing to patients’ health.”

## Implications for Public Health

This case study examined implementation of an intervention dedicated to closing preventive care gaps between recommended best practices and care actually delivered. Well-recognized change models, such as Kotter’s 8-Step Process for Leading Change, provide a systematic approach to guide a health clinic through elements needed for sustainable change, from a focus solely on emergent or chronic care delivery to a prevention focus. Inclusion of all of Kotter’s steps ensures that appropriate leaders in the organization guide such a change, that personnel involved in the change understand its purpose, and that the project is managed to the point of anchoring the change in the organization’s culture.

Although our evaluation had strengths, it also had limitations. Kotter’s model is most often used to guide large-scale change (6), and our project, although large for the White House Clinics, was small compared to other projects guided by the model. Leadership and staff members did not use the model proactively during the POE implementation planning process, and we did not assess validity and reliability of the model before using it in our case study. However, we found a strong connection between steps 1 through 7 of the change model and the implementation steps taken by White House Clinics. Step 8, anchoring new approaches in an organization’s culture, was an area for improvement; this step is often problematic for organizations implementing new initiatives (6,9). In addition, the findings of the case study might not be generalizable to other FQHCs or similar clinical settings.

Despite these limitations, application of such organizational change frameworks could be particularly useful for clinical settings, such as FQHCs, that historically do not have the same resources as large health care organizations and that serve a substantial proportion of medically underserved patients. Use of change-management approaches can help guide these organizations through strategic implementation and sustainability of an evidence-based intervention such as POE. Thus, the change-management approach not only serves to improve clinical workflows of the organization but also serves to improve patient outcomes and subsequently population health.
